# Compositional studies and biological activities of some mash bean (*Vigna mungo* (L.) Hepper) cultivars commonly consumed in Pakistan

**DOI:** 10.1186/0717-6287-47-23

**Published:** 2014-05-30

**Authors:** Muhammad Zia-Ul-Haq, Shakeel Ahmad, Shazia Anwer Bukhari, Ryszard Amarowicz, Sezai Ercisli, Hawa ZE Jaafar

**Affiliations:** The Patent Office, Karachi, Pakistan; Department of Agronomy, Bahauddin Zakariya University, Multan, 60800 Pakistan; Department of Applied Chemistry and Biochemistry, Government College University, Faisalabad, Pakistan; Institute of Animal Reproduction and Food Research of the Polish Academy of Sciences, Tuwima Str. 10, 10-747 Olsztyn, Poland; Agricultural Faculty, Department of Horticulture, Ataturk University, Erzurum, Turkey; Department of Crop Science, Faculty of Agriculture, 43400 UPM Serdang, Selangor Malaysia

**Keywords:** Nutrients, Antioxidant potential, Mash bean cultivar, Pakistan

## Abstract

**Background:**

In recent years, the desire to adopt a healthy diet has drawn attention to legume seeds and food products derived from them. Mash bean is an important legume crop used in Pakistan however a systematic mapping of the chemical composition of mash bean seeds is lacking. Therefore seeds of four mash bean (*Vigna mungo* (L.) Hepper, family *Leguminoseae*) cultivars (NARC-Mash-1, NARC-Mash-2, NARC-Mash-3, NARC-Mash-97) commonly consumed in Pakistan have been analyzed for their chemical composition, antioxidant potential and biological activities like inhibition of formation of advanced glycation end products (AGE) activity and tyrosinase inhibition activity.

**Results:**

The investigated cultivars varied in terms of biochemical composition to various extents. Mineral composition indicated potassium and zinc in highest and lowest amounts respectively, in all cultivars. The amino acid profile in protein of these cultivars suggested cysteine is present in lowest quantity in all cultivars while fatty acid distribution pattern indicated unsaturated fatty acids as major fatty acids in all cultivars. All cultivars were found to be rich source of tocopherols and sterols. Fourier transform infrared spectroscopy (FTIR) fingerprints of seed flour and extracts indicated major functional groups such as polysaccharides, lipids, amides, amines and amino acids. Results indicated that all investigated cultivars possessed appreciable antioxidant potential.

**Conclusions:**

All cultivars are rich source of protein and possess sufficient content of dietary fiber, a balanced amino acid profile, low saturated fatty acids and antioxidant capacity that rationalizes many traditional uses of seeds of this crop besides its nutritional importance. The collected data will be useful for academic and corporate researchers, nutritionists and clinical dieticians as well as consumers. If proper attention is paid, it may become an important export commodity and may fetch considerable foreign exchange for Pakistan.

## Background

Mash bean (*Vigna mungo* (L.) Hepper) family *Leguminoseae* locally known as *sabut maash*, is a highly praised legume in Pakistan due to its dieto-therapeutic importance. Seeds are used in culinary dishes since primeval. The seeds are eaten after cooking. Seeds are the chief constituent of many traditional products like *wari, papad*, *idli*, *dosa, halwa* and *imrati*[1]. The seeds are well-known due to their therapeutic and nutritional potential.

The roots are narcotic and diuretic and are used for treating nostalgia, abscess, aching bones, dropsy, cephalgia and inflammation. The seeds are emollient, astringent, thermogenic, diuretic, aphrodisiac, nutritious, galactogauge, appetizer, laxative, styptic and nervine tonic. They are useful in treating scabies, leucoderma, gonorrhea, pains, epistaxis, piles, asthma, heart trouble, dyspepsia, anorexia, strangury, constipation, haemorrhoids, hepatopathy, neuropathy, agalactia, schizophrenia, hysteria, nervous debility, partial paralysis, facial paralysis and weakness of memory. Seeds are believed as spermatopoetic, and used for treating erectile dysfunction and premature ejaculation. Seeds are used for lengthening the hair, keeps them black and curing dandruff. Hot aqueous extracts of the leaves are used in the treatment of brain disorders, stomach, jaundice, rheumatic pain and inflammatory disorders. Seeds are considered fattening and flour made from seeds is excellent substitute for soap, leaving the skin soft and smooth and used in cosmetics in preparation of facial mask [2–6].

The mash bean occupies an important position in agriculture system of Pakistan and is grown annually on area of 27.6 thousand hectares with annual production of 13.6000 tonnes with 493 kg/ha as average yield [7]. It is grown all over the country, but its cultivation is concentrated mainly in Punjab, the major mash production province. It is the least researched crop among pulses in Pakistan as is apparent from scarcity of literature on it and as a result its area of cultivation and production are decreasing gradually [8].

The food industry globally is searching functional foods, nutraceuticals and botanicals to meet demand of consumers for natural, immunity-boosting and health-promoting plant based food products. To our knowledge, there is no study indicating chemical composition and antioxidant potential of seeds of mash bean cultivar indigenous to Pakistan. As part of our research studies to investigate the biochemical composition and antioxidant capacity of indigenous flora of Pakistan [9–13] this study has been conducted to determine the chemical composition, antioxidant activity and biological activities of seeds of mash bean cultivars.

## Results and discussion

Composition and contents of various constituents and components like various bioactive constituents and secondary metabolites, fixed and essential oil, fatty acids, tocopherol and sterol profile, mineral, amino acid, vitamin, protein and carbohydrate contents present in a food commodity like seed, fruit, vegetable, spice, grain or any other product derived from them varies depending upon many factors like plant variety, agronomic practices utilized in cultivation, stage of collection and geological and climatic conditions of area from where that food commodity or plant part (seed or fruit) is collected, and the method employed for its determination. So there is need to establish food composition database on regional and country level for various food commodities for various regions and countries respectively. Previously our research group has compiled compositional and nutritional information on various other legumes like chickpea, pea, cowpea, lentil and mung bean. In current study we have determined biochemical composition, their impact on health as well biological activities of a less-researched legume crop i.e. mash bean.

The data on the proximate composition is summarized in Table 1. The observed range for protein was 24.62% for NARC-Mash-97 to 25.48% for NARC-Mash-2 Mash 2. The crude fiber content ranged from 4.25% to 5.09%. The range observed for fat content was between 1.80 and 2.25% while carbohydrates ranged from 53.43% to 55.55%. The high carbohydrate contents present in mash bean seeds indicate its potential use as a prime source of energy to prevent marsamus in infants especially. Like other legumes, its seeds are also rich in protein, contain sufficient amount of dietry fibre and lesser amount of oil. The results are in partial agreement to those reported earlier for mash bean [14, 15] and other legumes [9–13]. Regular intake of dietry fibre is associated with low chances of cardiovascular disease, obesity, certain cancers and diabetes. High dietry fiber contents may be responsible for its traditional use as anti-cancer food. Since dietry fibre containing foods are used in bakery products, it also indicates its potential use in bakery and pastry products.

**Table 1:** **Proximate composition (%) of seeds of mash bean cultivars**

Component	NARC-Mash-1	NARC-Mash-2	NARC-Mash-3	NARC-Mash-97
Crude protein	27.91 ± 1.71^a^	26.48 ± 1.66^b^	25.07 ± 1.60^c^	28.60 ± 1.72^a^
Total lipids	5.13 ± 0.05^b^	6.00 ± 0.05^a^	5.80 ± 0.09^a^	6.22 ± 0.09^a^
Carbohydrates	56.55 ± 1.82^a^	54.81 ± 1.73^b^	58.13 ± 1.10^a^	54.81 ± 1.75^b^
Crude fiber	5.44 ± 1.7^b^	6.84 ± 1.60^a^	4.25 ± 1.20^b^	5.11 ± 1.60^b^
Ash	4.97 ± 0.19^b^	5.87 ± 0.18^b^	6.72 ± 0.19^a^	5.26 ± 0.18^b^

Values in the same row having different letters differ significantly with least significant difference (LSD) at probability (p < 0.05).

**Figure 1:**
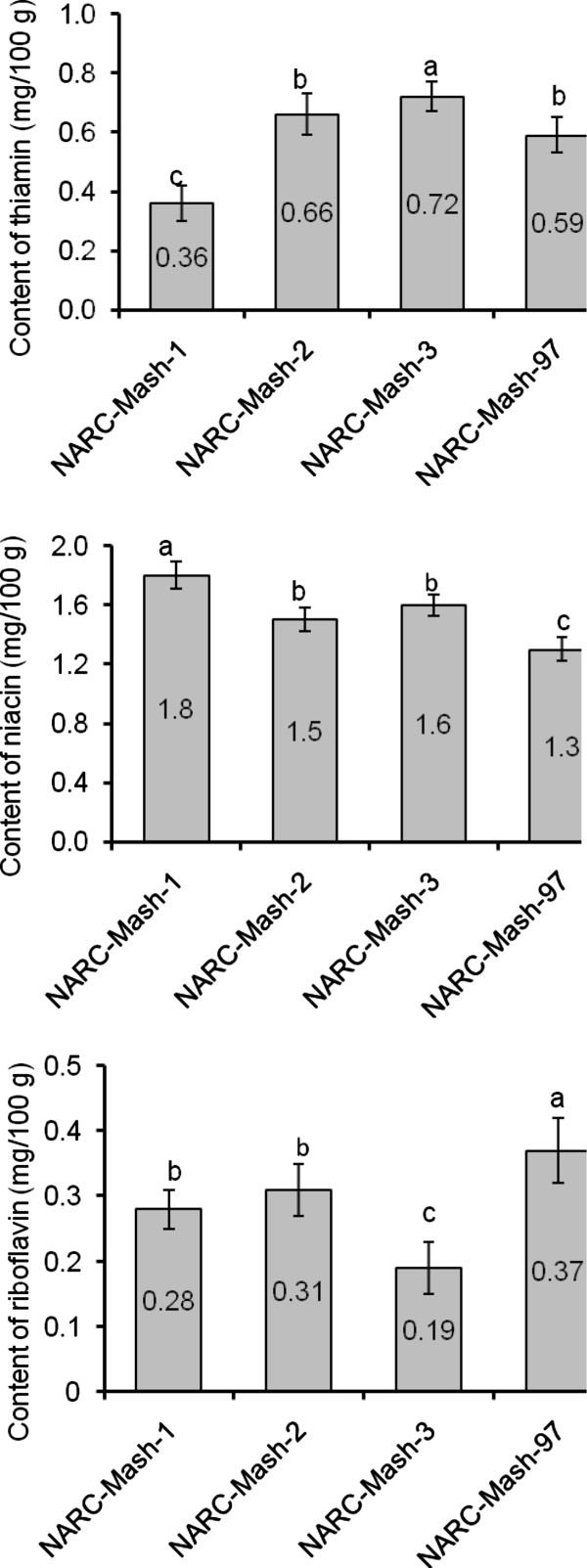
**Vitamin content (mg/100 g) of seeds of mash bean cultivars.**

The data of vitamin contents is summarized in Figure 1. Niacin content was highest in NARC-Mash-1 (1.80 ± 0.07 mg/g) while NARC-Mash-3 had lowest content of riboflavin (0.19 ± 0.19 mg/g). Regarding vitamin contents of seeds of mash bean, niacin was present in higher concentration among all cultivars. As there is no report available on vitamin contents of mash bean, so vitamin contents cannot be compared to previous results. However the vitamin contents are in close proximity to that of *Pisum sativum* as per our previous studies [16]. High contents of niacin are good from medical point of view as this water-soluble vitamin is excreted by urine from human body and its continuous supply by eating mash bean seeds will complete its deficiency. Various agro-geo-climatological conditions affect vitamin contents in legume seeds.

Mineral contents (Table 2) indicated potassium as major mineral from 1599.82 ± 1.74 mg/100 g in NARC-Mash-97 to 1646.01 ± 0.92 mg/100 g in NARC-Mash-3. Phosphorus ranked second in quantity from 439.79 ± 0.42 and 500.17 ± 1.85 in same varieties. Zinc was present in lowest content (1.94 ± 0.76 mg/100 g) in NARC-Mash-97. All cultivars contained sufficient contents of potassium, phosphorus and copper. It is perhaps this high potassium content that makes it an aphrodisiac. The high content of potassium is useful for patients who use diuretics to manage hypertension and there is unnecessary seepage of potassium from their body fluids. The low content of sodium compared to potassium led to a low sodium: potassium ratio, which is favorable from nutritional point of view, as foods with low Na:K ratio are linked with lower frequency of blood hypertension. Na:K ratio is from 0.14 to 0.17 in NARC-Mash-1 and NARC-Mash-3 respectively. For prevention of high blood pressure, Na/K ratio of less than one is suggested. This may explain the rationale behind the traditional use of its seeds in managing hypertension. Low Ca:P ratio leads to loss of Ca in the urine more than normal amount, so Ca concentration in bones is reduced. Food is considered “poor” if Ca:P ratio is less than 0.5 and “good” if it is above one. In present study, Ca:P ratio ranged from 0.78 to 1.00 in NARC-Mash-2 and NARC-Mash-1 respectively indicating regular consumption of mash bean seeds will serve as fine source of calcium for formation of bones. High levels of calcium are required during growth, gravidity and lactation of animals [13]. The results are in par to those already reported for mash bean elsewhere [17, 18]. It is well-known that mineral contents of plant and crops parts like fruit and seeds depend on cultivars, collection time and maturity stage, climatological conditions, agronomic practices like type of fertilizer and water as well as selectivity, acceptability and intake of minerals by crops and plants. These results suggested that mash bean may provide adequate quantity of minerals to meet the mineral requirements of human body [19].

**Table 2:** **Mineral content (mg/100 g) of seeds of mash bean cultivars**

Minerals	NARC-Mash-1	NARC-Mash-2	NARC-Mash-3	NARC-Mash-97
Phosphorus	461.24 ± 0.22^c^	480.47 ± 3.02^b^	500.15 ± 2.91^a^	440.90 ± 0.80^d^
Potassium	1603.39 + 1.66^b^	1638.88 ± 2.86^a^	1646.11 ± 3.17^a^	1600.03 ± 2.61^b^
Sodium	227.01 ± 4.55^c^	244.90 ± 1.41^b^	284.08 ± 2.01^a^	261.33 ± 1.79^a^
Calcium	462.90 ± 2.07^a^	375.01 ± 3.66^b^	485.38 ± 1.14^a^	394.19 ± 2.04^b^
Magnesium	263.83 ± 3.56^a^	239.70 ± 1.36^b^	208.45 ± 1.21^c^	221.77 ± 1.18^c^
Iron	5.89 ± 0.25^b^	6.14 ± 0.21^b^	6.38 ± 0.18^a^	6.55 ± 0.33^a^
Manganese	2.39 ± 2.07^b^	3.27 ± 0.05^a^	3.32 ± 0.11^a^	3.22 ± 0.18^a^
Zinc	2.40 ± 0.14^a^	2.28 ± 0.12^a^	2.50 ± 0.22^a^	1.94 ± 0.76^b^
Copper	3.92 ± 0.47^b^	4.03 ± 0.83^b^	4.26 ± 0.66^a^	4.51 ± 0.34^a^
Na/K	0.14	0.15	0.17	0.16
Ca/P	1.00	0.78	0.97	0.89

Values in the same row having different letters differ significantly with least significant difference (LSD) at probability (p < 0.05).

A protein-rich diet is not a guarantee to fulfill the requirements of the amino acids, a human body needs. A balanced protein diet should comprise all amino acids in sufficient amount and essential and non-essential amino acid ratio denotes the nutritional quality of protein. Glutamic acid (19.19 ± 0.62 to 21.49 ± 0.07 g/100 g) and aspartic acids (11.53 ± 0.11 to 13.20 ± 0.27 g/100 g) were present in highest amount in all cultivars. Except tryptophan and S-containing amino acids, all essential amino acids are present in sufficient amounts in all analyzed cultivars as is evident by data (Table 3). Most amino acids derived from plant sources are believed to possess antimicrobial, anti-inflammatory, immune-stimulating and antioxidant properties besides their role in nutrition. Results are comparable to those of previous studies on mash bean amino acids [17]. The deficient amino acids can be acquired by including large quantity of mash bean in diet, or by taking mash bean as well as other legumes.

**Table 3:** **Percentage composition of amino acids in seeds**

Amino acid	NARC-Mash-1	NARC-Mash-2	NARC-Mash-3	NARC-Mash-97
Alanine	4.63 ± 0.17^b^	5.20 ± 0.07^a^	4.35 ± 0.05^c^	4.17 ± 0.21^c^
Arginine	6.03 ± 0.27^c^	6.30 ± 0.04^b^	6.53 ± 0.03^a^	6.64 ± 0.15^a^
Aspartic acid	13.20 ± 0.27^a^	12.40 ± 0.08^b^	11.98 ± 0.07^bc^	11.53 ± 0.11^c^
Cystine	0.75 ± 0.29^b^	0.90 ± 0.04^a^	0.45 ± 0.03^c^	0.72 ± 0.23^b^
Glutamic acid	21.07 ± 0.65^a^	21.49 ± 0.07^a^	20.44 ± 0.09^b^	19.19 ± 0.62^c^
Glycine	4.39 ± 0.12^a^	4.61 ± 0.05^a^	3.73 ± 0.03^b^	4.34 ± 0.24^a^
Histidine	2.36 ± 0.31^b^	2.13 ± 0.02^b^	3.21 ± 0.01^a^	3.26 ± 0.26^a^
Isoleucine	4.48 ± 0.17^a^	4.37 ± 0.07^a^	3.79 ± 0.05^b^	4.25 ± 0.09^a^
Leucine	8.89 ± 0.12^a^	7.31 ± 0.03^b^	7.79 ± 0.04^b^	7.54 ± 0.45^b^
Lycine	4.19 ± 0.88^d^	7.69 ± 0.01^a^	6.90 ± 0.08^b^	5.07 ± 0.74^c^
Methionine	1.92 ± 0.74^a^	1.12 ± 0.05^b^	1.42 ± 0.09^b^	1.29 ± 0.29^b^
Phenylalanine	5.59 ± 0.18^a^	4.88 ± 0.06^b^	5.80 ± 0.07^a^	5.67 ± 0.12^a^
Proline	4.30 ± 0.21^b^	3.69 ± 0.03^c^	5.01 ± 0.01^a^	4.20 ± 0.08^b^
Serine	5.18 ± 0.30^a^	5.31 ± 0.05^a^	4.14 ± 0.08^c^	4.78 ± 0.07^b^
Threonine	3.95 ± 0.35^b^	3.80 ± 0.04^b^	4.50 ± 0.03^a^	3.99 ± 0.28^b^
Tryosine	1.01 ± 0.14^c^	1.70 ± 0.09^b^	2.80 ± 0.02^a^	3.15 ± 0.28^a^
Tryptophan	2.97 ± 0.19^a^	2.40 ± 0.06^c^	2.67 ± 0.02^b^	2.92 ± 0.07^a^
Valine	5.09 ± 0.11^a^	4.80 ± 0.08^b^	4.94 ± 0.04^a^	5.08 ± 0.04^a^

Values in the same row having different letters differ significantly with least significant difference (LSD) at probability (p < 0.05).

**Figure 2:**
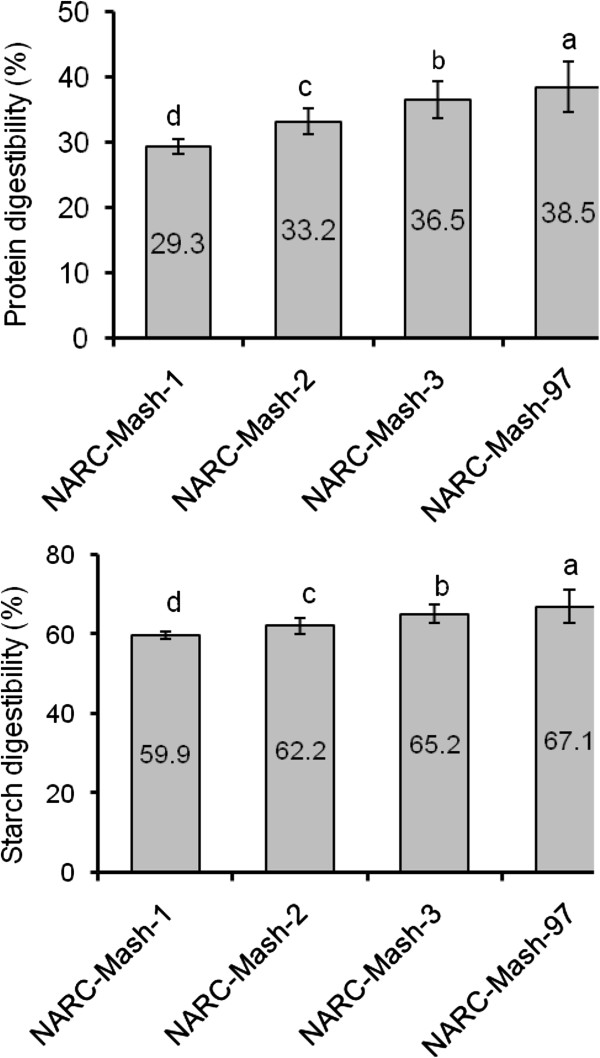
***In-vitro*** **protein and starch digestibility (%) of mash bean seeds.**

Besides amino acid composition, protein digestibility is crucial for determining the protein quality. *In-vitro* protein digestibility data (Figure 2) suggested that values are lowest in NARC-Mash-1 (29.30 ± 0.82%) and highest in NARC-Mash-97 (38.53 ± 0.21%) while starch digestibility was 59.93 ± 0.17 to 67.09 ± 0.02 for same cultivars. Protein digestibility was below 50 percent while starch digestibility was above 50 percent in all analyzed cultivars. A significant variation has been observed for protein digestibility of legume seeds previously for mash bean and other legumes [16, 17]. The sensory, textural and nutritional characteristics of products made from legumes are due to various functional properties of proteins. Anti-nutritional components like tannins, phytates and trypsin inhibitors, and structural distinctiveness of storage proteins slow down the digestibility of legume proteins. Treatments like roasting; autoclaving and cooking may be utilized to increase the legume proteins digestibility. In vitro starch digestibility values are close to those reported earlier [20]. Since legume starches generally contain more amylase, therefore these are less digestible. This low digestibility is useful as it decreases release of glucose in blood and so is helpful for patients suffering from diabetes. It may be reason of prescribed use of mash bean for diabetic patients by traditional healers. The low-digestibility however may be managed by utilization of legume seeds along with husk since dietary fibre present in husk will decrease the transit time in intestines and will help in bowel motility.

Fatty acids profile determines the oil quality of seeds or fruits or any other part of plant and products derived from them. Fatty acids profile of oil of seeds of investigated cultivars is summarized in Table 4. All cultivars were found to be rich source of α-linolenic acid (49.52 ± 0.09 to 51.80 ± 0.03%) and oleic acid (26.62 ± 0.07 to 27.34 ± 0.25%). Bulk of the oil consisted of unsaturated fatty acids for all cultivars. The results are comparable to previously published works for low-oil bearing legumes in general [21] and for mash bean in particular [22]. Saturated fatty acids were a small percentage of total fatty acids present. There is reduced risk of cholesterol-related heart diseases by consuming oils containing more unsaturated fatty acids. However since oil content is very low in seeds therefore it cannot be considered as commercial source of vegetable oil.

**Table 4:** **Fatty acid composition (%) of oil of mash bean seeds**

Fatty acid	NARC-Mash-1	NARC-Mash-2	NARC-Mash-3	NARC-Mash-97
Palmitic acid	11.31 ± 2.20^b^	10.99 ± 1.99^c^	11.23 ± 1.87^b^	12.09 ± 1.58^a^
Stearic acid	2.09 ± 0.63^b^	2.70 ± 0.24^a^	2.89 ± 0.43^a^	2.17 ± 0.77^b^
Behenic acid	0.99 ± 0.14^a^	1.00 ± 0.29^a^	0.87 ± 0.22^a^	0.93 ± 0.30^a^
Oleic acid	26.62 ± 0.07^b^	26.74 ± 0.15^b^	27.34 ± 0.25^a^	26.65 ± 0.35^b^
Linoleic acid	07.19 ± 4.47^b^	08.93 ± 5.11^a^	07.08 ± 3.74^b^	08.64 ± 3.87^a^
α-Linolenic acid	51.80 ± 0.03^a^	49.64 ± 0.06^a^	50.59 ± 0.05^a^	49.52 ± 0.09^a^

Values in the same row having different letters differ significantly with least significant difference (LSD) at probability (p < 0.05).

Data about tocopherol composition is summarized in Table 5. Despite differences, *γ*-tocopherol contents were present in highest quantity in all cultivars while considerable contents of *δ*-tocopherol followed *α-*tocopherol were also noted. Oil of seeds of all mash bean cultivars studied contained all major tocopherols. Like many other traits, no previous report is present regarding tocopherol and sterol contents of mash bean seeds. However all values are close to those reported for Indian mash bean seeds [22]. Since naturally occurring tocopherols are used for oils and fats stabilization against oxidative degradation, it suggests their usage in pharmaceutical, biomedical, and nutritional products.

**Table 5:** **Tocopherol content (mg/100 g) in oil of seeds of mash bean cultivars**

Tocopherols	NARC-Mash-1	NARC-Mash-2	NARC-Mash-3	NARC-Mash-97
α-Tocopherol	3.04 ± 0.89^b^	2.97 ± 0.55^b^	3.49 ± 0.17^a^	3.17 ± 0.34^b^
γ-Tocopherol	722.09 ± 2.17^a^	724.34 ± 4.13^a^	722.21 ± 1.16^a^	720.33 ± 2.01^a^
δ-Tocopherol	16.69 ± 3.3^b^	16.18 ± 4.2^c^	17.14 ± 2.66^a^	17.12 ± 4.0^a^

Values in the same row having different letters differ significantly with least significant difference (LSD) at probability (p < 0.05).

Sterol profile is summarized in Table 6. Substantial amounts of campesterol, avenasterol and stigmasterol were found in oils of seeds of all four cultivars. The main sterol in oil of seeds of all investigated mash bean cultivars was β-sitosterol which is in agreement with previous studies for low-oil bearing legumes like chickpea, mungbean, cowpea, *Albizia lebbeck* and *Acacia leucophloea* in general [10, 12, 13, 21] and for mash bean in particular [23]. Various agro-geo-climatological factors as well as solvent used for extraction of oil are believed to be responsible for the distribution of tocopherols and sterols in oils extracted from plant parts. Sitosterol, campestrol and stigmasterols have been observed to be major sterols in oils from most of plants belonging to family *Leguminosae*[10, 12, 13, 21].

**Table 6:** **Sterol content (mg/100 g) in oil of seeds of mash bean cultivars**

Sterols	NARC-Mash-1	NARC-Mash-2	NARC-Mash-3	NARC-Mash-97
β-Sitosterol	56.5 ± 0.2^a^	55.1 ± 0.6^a^	55.1 ± 0.61^a^	56.1 ± 0.51^a^
Stigmasterol	34.0 ± 0.4^a^	34.4 ± 0.8^a^	34.4 ± 0.8^a^	33.4 ± 0.1^b^
Δ^5^- Venasterol	4.00 + 0.22^b^	3.64 + 0.38^b^	3.64 + 0.38^b^	4.51 + 0.12^a^
Stigmastanol	2.66 + 0.05^c^	3.79 + 0.12^a^	3.79 + 0.12^a^	3.21 + 0.38^b^
Δ^7^- avenasterol	1.01 ± 0.17^a^	1.05 ± 0.69^a^	1.05 ± 0.69^a^	1.09 ± 0.43^a^
Campesterol	0.87 ± 0.40^b^	0.98 ± 0.80^a^	0.98 ± 0.80^a^	0.66 ± 0.18^c^
Unidentified	1.00 ± 0.03^a^	1.00 ± 0.27^a^	1.00 ± 0.27^a^	1.00 ± 0.13^a^

Values in the same row having different letters differ significantly with least significant difference (LSD) at probability (p < 0.05).

FTIR-fingerprints, give a quick check of identification, classification and discrimination of food samples by providing a general outline of pattern and trends indicating presence of various chemical compounds in samples. FTIR spectrum of mash bean seed powder indicated the presence of various types of aliphatic and aromatic compounds, especially carboxylic acids, esters, alkyl halides and nitro compounds. The presence of carboxylic acids is indicated by peaks at 2929.40 (O-H stretching), 1249.94 cm^−1^ (C-O stretching). The peak at 1728.04 corresponds to C = O stretching frequency of aldehydic group. Unsaturated compounds presence is indicated by the peak at 1658.57 cm^−1^ (C = C stretching, alkene) and 1556.41 cm^−1^ (C-C stretching, aromatic compounds). Saturated compounds presences is shown by the peak at 1450.17 cm^−1^ (C-H bending, alkane). Nitro compounds and aromatic amine presence is indicated by the peaks at 1343.93 and 1319.41 cm-1. The peaks at 1160.05 and 1074.23 cm-1 showed the presence of aliphatic amines. Alkyl halides presence is pointed out by the peak at 849.49 cm^−1^.

For mash bean extract, the highly intensified OH region with intensified shoulder peak of amine group was present. A new peak in the region of 1700–1800 was observed which may be attributed to presence of ester. Saturated compounds presence is indicated by the peaks at 2925.31, 2859.93 (C-H stretching, alkanes) and 1384.79 cm^−1^ (C-H rocking, alkane). Carboxylic acid presences is confirmed by the peaks at 3023.38 (O-H stretching), 1736.21 and 1695.35 cm^−1^ (C = O stretching). Nitro compounds presences are indicated by the peak at 1515.55 cm^−1^ (N-O asymmetric stretching). Primary aliphatic amines presences is indicated by peaks at 1466.52 (N-H bending) and 1221.34 cm^−1^ (C-N stretching). Aromatic compounds presence is shown by peak at 1466.52 cm^−1^ (C-C stretching in ring). The observed bands for amines, amides, amino acids confirmed the presence of proteins, whereas presence of other bio-molecules like carboxylic acids, carbohydrates and oil was indicated by other absorption bands. Bhat et al. [24] and Zia-Ul-Haq et al. [25] have reported previously similar functional groups in *Gnetum gnemon* L. and *Pisum sativum* L. respectively.

**Figure 3:**
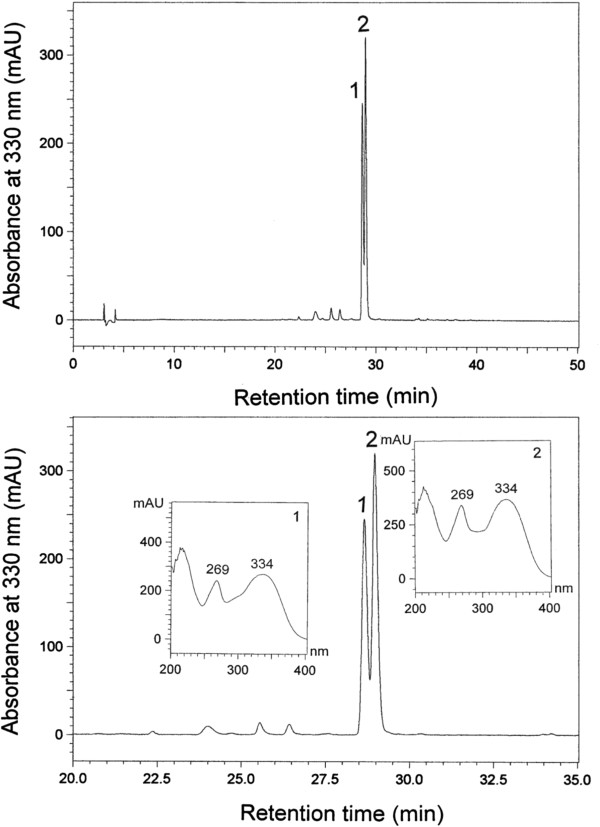
**HPLC spectra of mash bean (NARC-Mash-97) seed extract.**

Especial attention is being given to the identification of phenolic acids, flavonoids and tannins from extracts of legume seeds. Total phenolic content (TPC, mg GAE/g) of seed extracts from selected mash bean cultivars are presented in Table 7. The TPC was observed in highest amount in NARC-Mash-97 (86 mg GAE/g), whereas the lowest TPC was noted for NARC-Mash-1 (75 mg GAE/g). The total flavonoids contents (TFCs) and condensed tannins (CTC) were expressed in catechin equivalents (CAE/g). The cultivars differed significantly (*P <* 0.05) in TFCs and CTCs. The chromatograms (RP-HPLC) of extracts of seeds of mash bean were recorded at 330 nm and two dominant peaks (1–2) with a retention times of 28 and 28.8 min respectively (Figure 3), were observed. The spectra (UV) of both compounds (peaks 1–2) displayed maxima at 269 and 334 nm. Compounds **1**, and **2** were identified as chlorogenic acid and caffeic acids when compared with standards run simultaneously. The mash bean extracts investigated in this study were characterized by several times higher content of flavonoids and condensed tannins when compared to desi chickpea, kabuli chickpea, lentil, cowpea, *Albizia lebbeck* and *Acacia leucophloea* varieties [11–13, 25, 26]. Presence of higher contents of various phenolic compounds was noted in extracts (Table 8). Various phenolic acids have been identified earlier in extracts from other legumes like chickpea, cowpea and pea [11, 25, 26]. Consumption of phenolic-rich foods is associated with low risk of several chronic diseases such as cardiovascular disease, ageing, cancer, neurodegenerative disease and Alzheimer disease as is evident from various epidemiological studies which highlights importance of presence of ample contents of phenolic acids noted in mash bean extract.

**Table 7:** **Total phenolic contents, total flavonoid contents and condensed tannin contents in extracts of seeds of mash bean cultivars**

Cultivar	Total phenolic contents	Total flavonoid contents	Condensed tannin contents
NARC-Mash-1	75.91 + 2.72^c^	51.78 + 1.85^b^	86.79 + 1.56^b^
NARC-Mash-2	79.33 + 1.52^b^	47.11 + 2.47^c^	89.14 + 1.11^b^
NARC-Mash-3	82.22 + 1.36^b^	42.66 + 1.81^d^	93.68 + 1.65^a^
NARC-Mash-97	86.99 + 1.19^a^	55.73 + 1.92^a^	79.20 + 1.77^c^

Values in the same row having different letters differ significantly with least significant difference (LSD) at probability (p < 0.05).

**Table 8:** **Content of two main phenolic compounds in the extracts and seeds of mash bean cultivars**

Cultivar	Compound 1	Compound 2	Compound 1	Compound 2
	(mg/g extract)	(mg/g extract)	(mg/g fresh seeds)	(mg/g fresh seeds)
NARC-Mash-1	4.09 ± 0.20^d^	5.22 ± 0.26^d^	0.39 ± 0.02^c^	0.50 ± 0.02^c^
NARC-Mash-2	5.94 ± 0.30^a^	8.09 ± 0.40^a^	0.55 ± 0.03^a^	0.74 ± 0.04^a^
NARC-Mash-3	5.48 ± 0.27^b^	7.35 ± 0.37^b^	0.48 ± 0.02^b^	0.65 ± 0.03^b^
NARC-Mash-97	5.07 ± 0.25^c^	6.94 ± 0.35^c^	0.44 ± 0.02^b^	0.44 ± 0.02^c^

Values in the same row having different letters differ significantly with least significant difference (LSD) at probability (p < 0.05).

The human body has several mechanisms to shield bio-molecules against damage caused by reactive oxygen and nitrogen species. However, the instinctive protection may not be adequate to counter the rigorous or continuous oxidative stress. Hence, certain amounts of exogenous antioxidants are frequently required to maintain sufficient antioxidants level to balance the reactive nitrogen and oxygen species-pressure in the human body. Scientists are exploring antioxidants from natural sources like legume seeds as these are natural, cost effective and without side effects. The scavenging activity of mash bean extracts was expressed by antiradical assays against DPPH^•^ and ABTS^•+^ assay as well as by FRAP and reducing power assays as shown in Table 9. DPPH values of mash bean varieties ranged from 34.72 in NARC-Mash-2 to 39.49 μmol Trolox/g in NARC-Mash-3. Sufficient scavenging of DPPH radical was observed by extracts. It indicates that antioxidants present in extracts quench free radicals by donating them hydrogen atoms thereby converting them to non-toxic species. Although assessment of antiradical activity of an extract by DPPH protocol is fast and trouble-free, it usually has a relatively small linear reaction range therefore antiradical activity against ABTS^•+^ was measured. The ABTS^•+^ scavenging data indicated that the extracts may scavenge free radicals by hydrogen/electron donation mechanism and may protect biomatrices from oxidative degradation resulting from free radicals. Substantial antiradical activity for DPPH and ABTS^•+^ was observed with same order of scavenging in both protocols. It was noted that reducing potential of extracts increased with increasing amount of extracts. Butylated hydroxanisole was used as standard to compare the reducing power of extracts. Mechanistic studies indicate that antioxidant potential of extracts is closely linked with their reducing power. The results were close to reported earlier [27–29].

**Table 9:** **Antioxidant capacity of extracts of seeds of mash bean cultivars**

Cultivar	Reducing power	DPPH^•^ scavenging capacity	FRAP	ABTS scavenging capacity
	(mg/g)	(μmol Trolox/g)	(mmol Fe^2+^/g)	(μmol trolox/g)
NARC-Mash-1	1.09 ± 0.18^a^	41. 64 ± 0.18^b^	12.81 ± 0.03^a^	33.81 ± 0.45^a^
NARC-Mash-2	0.87 ± 0.02^a^	34.72 ± 0.29^c^	11.70 ± 0.19^a^	27.09 ± 0.58^b^
NARC-Mash-3	1.02 ± 0.09^a^	39.49 ± 0.11^b^	9.65 ± 0.37^b^	29.74 ± 0.83^b^
NARC-Mash-97	0.95 ± 0.06^a^	46.56 ± 0.05^a^	13.76 ± 0.57^a^	35.93 ± 0.22^a^

Values in the same row having different letters differ significantly with least significant difference (LSD) at probability (p < 0.05).

The FRAP assay determines antioxidant activity of extracts as their potential to reduce ferric ions to ferrous ions. The FRAP values of the extracts of seeds of selected cultivars are presented in Table 9. Similar to that in DPPH^•^ analyses, high variations of FRAP values were observed and FRAP values of cultivars ranged from 9.65 mmol Fe^2+^/g in NARC-Mash-3 to 13.76 mmol Fe^2+^/g in NARC-Mash-97. Our results for FRAP are different from those reported earlier [27–29]. Antioxidant activity of the extracts of seeds of other plant like pea, cowpea, lentil, garden cress, capper and chickpea has been reported in several studies [25, 26, 30–32] by our research group.

**Figure 4:**
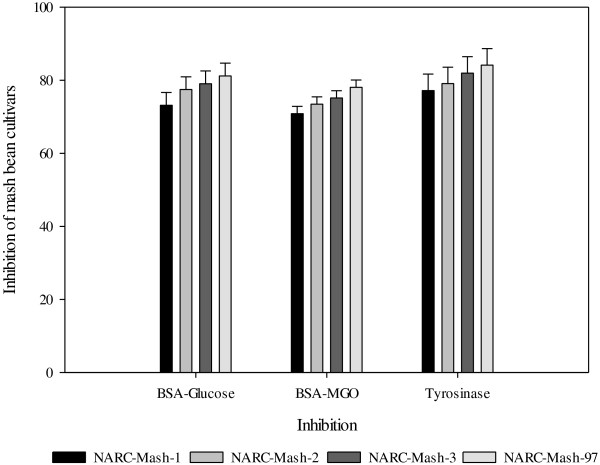
**Percentage inhibition of formation of advanced glycation end products (AGE) activity and tyrosinase inhibition by extracts of seeds of mash bean cultivars.**

It is generally believed that diabetes can be cured with more consumption of legumes however the mechanism behind this remained unexplored till now. The recent studies indicated that legumes cure diabetes by reducing AGE-formation. Advanced glycation end products (AGE) formation is increased in diabetes mellitus, so search for (AGEs)-inhibitor is a new approach in diabetes treatment. Two models used mostly for quantification of AGE-inhibtion of plant extracts are BSA-MGO and BSA-glucose models. In advanced glycation end (AGE) products inhibition activity, NARC-Mash-97 exhibited the highest inhibition (86.67%), followed by NARC-Mash-3 (74.84%) in BSA-glucose method. BSA-MGO inhibition model showed the same trend like that of BSA-glucose model (Figure 4). It is believed that phenolic compounds present in legume seeds inhibit the AGE-formation by inhibiting production of free radical during glycation process and subsequently inhibiting protein modification. The results (Figure 4) obtained in our study are in agreement with those reported previously for other legume seeds [33–38]. Same trend was observed in tyrosinase inhibition activity as was for AGE inhibition. Tyrosinase inhibition potential of extracts of seeds of mash bean may be ascribed to the presence of phenolic contents since hydroxyl groups present in various phenolic acids make a hydrogen bond at active site of the tyrosinase and as a result tyrosinase activity is decreased or stopped. Tyrosinase inhibitors have potential applications in food and cosmetic industry because they are used to stop or slow-down browning of various food commodities like fruits, vegetable and fisheries products and impart whitening effects to skin by stopping human skin hyper-pigmentation. The browning of food commodities leads to decrease in attractive appearance and loss of nutritional quality. It rationalized traditional use of mashbean in facial massages by indigenous communities and proves its anti- freckles, anti-wrinkling, anti-ageing and skin-whitening activity. For the first time tyrosinase inhibition activities of extracts of seeds of mash beans are being reported.

Statistically non-significant and very low correlations were found between different parameters viz. FRAP with BSA-G, BSA-MGO and TI; FRAP with BSA-MGO and TI at P < 0.05 (Table 10). TPC was having statistically no correlation with FRAP. Similarly, there was very low correlation between ABST and BSA-G. Medium correlations ranged between 0.28-0.55. Most of the correlations in this range were statistically non-significant except for DPPH and BSA-MGO (p < 0.05) and DPPH and TI (p < 0.05). Very high and positive correlations were found among certain variables like BSA-G and BSA-MGO, BSA-G and TI, BSA-MGO and TI, TPC and BSA-G, TPC and BSA-MGO and TPC and TI. The correlation between DPPH and FRAP was also statistically significant at p < 0.05, DPPH and ABST at p < 0.01, FRAP and ABST were also highly correlated (p < 0.01).

**Table 10:** **Correlation coefficient of total phenolics contents, DPPH, FRAP, ABTS BSA-MGO, BSA-Glucose and tyrosinase inhibition assay**

	TPC	DPPH	FRAP	ABST	BSA-G	BSA-MGO	TI
TPC	-	0.499	0.087	0.282	0.981^b^	0.999^b^	0.992^b^
DPPH		-	0.603^a^	0.967^b^	0.321	0.513^a^	0.551^a^
FRAP			-	0.718^b^	−0.034	0.132	0.046
ABTS				-	0.092	0.303	0.330
BSA-G					-	0.977^b^	0.960^b^
BSA-MGO						-	0.987^b^
TI							-

^a^Correlation is significant at p < 0.05 level (2-tailed).

^b^Correlation is significant at p < 0.01 level (2-tailed).

## Conclusion

The results suggested mash bean seeds as a rich source of nutrients and extracts of seeds exhibited good antioxidant and biological activities. Seeds are rich source of protein and carbohydrate and good source of dietry fibre. These also contain ample amount of essential minerals like Ca, K, Na, Mg, Cu and Zn and various essential and non-essential amino acids. Seeds also have acceptable fatty acids, tocopherol and sterol profile. Various functional groups were detected in FTIR of seeds and extracts. Antioxidant results suggested them as rich source of phenolic acids, flavonoids and condensed tannin contents. The extracts indicated good tyrosinase and AGE-inhibition activity. These results suggest that mash bean seed may be used in food industry as functional food and nutraceutical as well as in cosmetic and pharmaceutical industry as ingredient of skin-whitening creams and as cure for diabetes respectively. The data obtained will be helpful for labeling of nutrients as well as for monitoring the quality and authenticity of foods containing mash bean in indigenous markets. Further investigations are necessary to evaluate the toxic effects (if any), to determine the ant-nutrients factors present and to understand mechanism of action of tyrosinase-inhibitory and AGE-inhibitory potential of extracts.

## Methods

### Material

Analytical grade solvents were used. All chemicals were were purchased from Sigma except where indicated. The seeds of four mash bean cultivars namely, NARC-Mash-1,NARC-Mash-2, NARC-Mash-3 and NARC-Mash-97 were procured from National Agricultural Research Centre, Islamabad (Pakistan). Seeds of four cultivars were stored in stainless-steel containers at 4°C prior to analysis.

### Proximate analysis

Proximate chemical analysis of seeds was carried out according to AOAC International methods as per our previous studies [39]. Results are shown in Table 1.

### Vitamin contents

Powdered sample (5 g) was steamed with concentrated H_2_SO_4_ (30 ml) for half an hour. After cooling, distilled H_2_O was added to this suspension to make its volume up to 50 ml and filtered. Basic lead acetate (60%, 5 ml) was added to this filtrate (25 ml). The pH was adjusted (9.5) and supernatant was collected after centrifugation. To this supernatant, concentrated H_2_SO_4_ (2 ml) was added. After 1 hr, this mixture was centrifuged and then ZnSO_4_ (5 ml, 40%) was added. The pH was adjusted (8.4) and supernant was collected after centrifugation. The pH of resulting supernatant was adjusted (7) and this was utilized as niacin extract. One ml of this extract was made 6 ml by distilled H_2_O; after addition of cyanogen bromide (3 ml) and shaking, aniline (4%, 1 ml) was added. After 5 min, yellow color formed was spectrophotometrically measured at 420 nm against blank and niacin contents were calculated by a standard graph [16]. Thiochrome method and fluorescence method were used for determination of thiamine and riboflavin contents respectively [40, 41] Figure 1.

### Minerals contents

A muffle furnace was used to incinerate seeds (450°C; 12 h) and the resulting samples were digested by acid mixture (nitric/perchloric;2:1). Na and K were estimated by taking aliquots from this digested material by flame photometer. Other minerals like Mn, Mg, Ca, Fe, Cu and Zn were estimated spectrophotometrically (AAS; Perkin-Elmer 5000) while phosphovanado-molybdate method was used to measure phosphorus contents. Standard solutions of known concentration were run concurrently to quantify the samples [31, 32] (Table 2).

### Amino acid analysis

HCl (6 M) was used to hydrolyze samples (300 mg) in an evacuated test tube (105°C; 24 h). Citrate buffer (pH 2.2) was used to dissolve the dried residue resulting from flash evaporation. Hitachi Perkin-Elmer (KLA 3B) amino acid analyzer was utilized to quantify amino acids by taking aliquots from above solution. After treatment with performic acid followed by hydrolysis (HCl), cystine and methionine were analyzed separately from same solution. Alkali hydrolysis (NaOH) method was used to measure tryptophan [22, 23] (Table 3).

### Protein and starch digestibility (*In-vitro*)

*In-vitro* digestibility of protein was evaluated enzymetically while starch digestibility was evaluated as starch hydrolyzed (%) out of total starch present in sample [42–44] (Figure 2).

### Fatty acid (FA) composition

Petroleum ether as solvent was used to extract oil from seeds by Soxhlet apparatus (6 hr) as per official AOCS method [39]. The fatty acid profile of oils obtained was evaluated by a method reported earlier [45]. Briefly, *n*-heptane (1 mL) was used to dissolve oil (1 drop), sodium methanolate (50 μL; 2 M) was added, and shaken in a closed tube (1 min). Water (100 μL) was added and the tube was centrifuged (4500 g; 10 min) and resulting aqueous phase was separated. To remaining heptane phase, HCl (50 μL; 1 M) was added, both phases were mixed for short period of time and resulting aqueous phase was discarded. After addition of sodium hydrogen sulphate (20 mg) and centrifugation (4500 g; 10 min), n-heptane phase was stored in a vial and inserted in a gas chromatograph (Varian 5890) having CP-Sil88 capillary column (ID: 0.25 mm, 100 m, film: 0.2 μm). The temperature setup was as follows: heated (155°C- 220°C; 1.5°C/min), isotherm (10 min); detector and injector (250°C), carrier gas (H_2_: 1.07 mL/min), split ratio of 1:50; detector gas (hydrogen: 30 mL/min). Peaks were computed with help of integration software and fatty acid methyl esters (%) were obtained as weight percent by direct internal normalization (Table 4).

### Tocopherol contents

Twenty five ml of n-heptane was mixed with oil (250 mg) and tocopherol contents were was analyzed by HPLC system (Merck-Hitachi), containing a pump (L-6000), a fluorescence spectrophotometer (Merck-Hitachi F-1000), excitation wavelength (295); emission wavelength (330 nm) and a D-2500 integration system; 20 μl of samples were inserted by a Merck 655-A40 autosampler in a dual phase HPC (Merck) having column column (25 cm × 4.6 mm) while flow rate was adjusted at 1.3 mL/min. Mobile phase used was n-heptane: tert-butyl methyl ether (99:1) [46] (Table 5).

### Sterol composition

The sterols were quantified by a gas chromatograph (Perkin Elmer model 8700), having flame-ionization detector (FID) and OV-17 capillary column (methyl phenyl polysiloxane coated; ID: 30 m × 2.25 mm, film: 20 μm). The column was operated isothermally (255°C) while temperature for injector and detector were 275 and 290°C, respectively. Carrier gas selected was extra pure nitrogen with 3 mL/min as flow rate. Sterols were recognized and quantified by comparing with a sterol standard mixture [21, 31] (Table 6).

### Extraction

The mash bean seeds were ground to flour by a mill (IKA Works Inc.) and were sieved (60-mesh). After maceration with 5 L solvent mixture of aqueous: methanol (80:20) for 15 days at room temperature and extracts were collected. The process was carried out three times. The resulting extracts were collected and filtered by filter paper. The extra solvent present was evaporated under reduced pressure by using a rotary evaporator. A thick gummy mass was obtained which was then dried in a dessicator and utilized for assessment of biological activities.

### FTIR of Mash bean powder and crude extract

**Figure 5:**
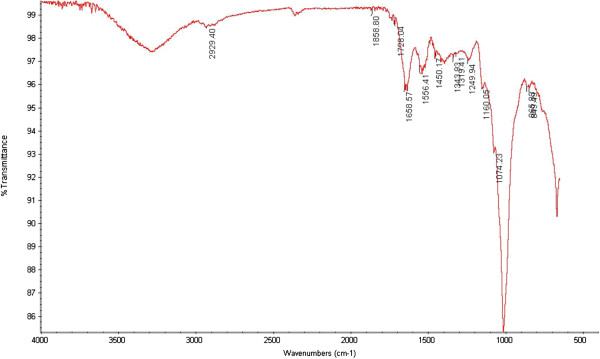
**FTIR spectrum of mash bean seed powder.**

**Figure 6:**
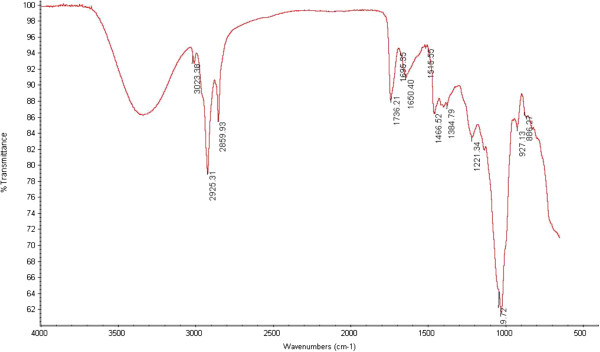
**FTIR spectra of Mash bean seed extract.**

Functional groups present in flour and extracts of seeds of mash bean cultivars were identified by FTIR spectroscopy (Perkin Elmer; UK) [24, 25] Figure 5 and 6.

### Total phenolic, flavonoid and condensed tannin contents (TPC, TFC, CTC)

Total phenolics were were estimated using the Folin and Ciocalteau’s phenol reagent [47] and results were reported as gallic acid equivalents [48, 49]. A previously reported method was used for estimation of flavonoids contents [50]. Condensed tannins were quantified by acidified vanillin reagent [51] and results were expressed as mg of CAE/g (Table 7).

### RP-HPLC

Phenolic acids were finger printed by using HPLC (Shimadzu Corp., Kyoto, Japan) fitted with a pre-packed LUNA C-18 column (4 × 259 mm, 5 μm) equipped with two LC-10 AD pumps, photodiode array detector (SPD-M 10), and a SCTL 10A system. Flow rate was adjusted at 1 mL/min and gradient elution of acetonitrile:water acetic acid (5:93:0) as solvent A and and acetonitrile:water acetic acid (40:58:2) as solvent B was used [52]. Samples were dissolved in methanol (10 mg/mL) while injection volume used was 20 μL. Separated compounds were measured at 330 nm Figure 3, Table 8.

### DPPH radical scavenging assay

Scavenging potential of extracts of mash bean seeds against DPPH^•^ was estimated by a previously reported method [53]. The absorbance of extracts (A_sample_) was measured spectrophotometrically (Shimadzu, Kyoto, Japan) at 517 nm and ethanol was used as blank. The extraction solvent (0.2 mL) after addition of DPPH^•^ was used as negative control (A_control_). Following equation was used to assess antiradical activity:

Calibration curve of Trolox was used to calculate results and indicated as micromoles of Trolox equivalent (*μ*mol Trolox/g) Table 9.

### Ferric reducing antioxidant power (FRAP) activity

FRAP assay was carried out to assess antioxidant activity [54]. Deionized water was used to dilute properly the sample solution to fit within the linearity range of Fe^2+^. The calibration curve of Fe^2+^ was used to calculate FRAP value as mmoles of Fe^2+^ equivalent (mmol Fe^2+^/g) Table 9.

### Reducing power

Reducing potential of investigated extracts was determined by a reported method [55]. Aliquotes (2.5 ml) of extracts dissolved in phosphate buffer (pH 6.6, 0.2 M) were mixed with C_6_N_6_FeK_3_ (10 mg/ml; 2.5 ml) and resulting solution was incubated (20 min; 50°C). To this reaction mixture, trichloroacetic acid (100 mg/ml solution; 2.5 ml) was added and centrifuged (1000 rpm; 10 min). The resulting supernant (2.5 ml) was mixed with an equal volume of H_2_O (distilled) and FeCl_3_ (1 mg/ml solution; 0.5 ml) was added. Spectrophotometer was used to measure absorbance at 700 nm against ascorbic acid Table 9.

### ABTS^•+^ scavenging assay

Scavenging activity of extracts of seeds was also evaluated against ABTS^•+^[56]. ABTS aqueous solution (5 mM) was passed from the oxidizing reagent (MnO_2_), on filter paper (Fisher Brand P8) to prepare ABTS^•+^. The solution was filtered from fisher membrane (0.2 mm) to remove extra MnO_2_. Phosphate buffered saline (5 mM; pH 7.4) was used to dilute extracts to an absorbance of approximately 0.700 (±0.020) at 734 nm. The extracts (1.0 mL) were added to ABTS^•+^ solution (5 mL), and the absorbance was measured after 10 min. The blank used was PBS Table 9.

### Evaluation of AGE inhibition activity

Inhibitory potential of mash bean extracts on the formation of advanced glycation end (AGE) products was determined by BSA-MGO and BSA-glucose models (Table 10). Briefly, BSA (5 g) and D-glucose (14.4 g) were dissolved in phosphate buffer (1.5 M; pH 7.4) to get a control solution containing D-glucose (0.8 M) and BSA (50 mg/mL). Two mL of this solution was incubated at 37°C (1 week) in the absence or presence of bean extracts (1 mL) in phosphate buffer. After one week, fluorescent intensity (excitation: 330 nm; emission: 410 nm) was measured. The BSA-MGO assay was performed as reported elsewhere.Briefly, MGO (31 μL) was mixed with BSA (40 mg) in phosphate buffer (pH 7.4; 0.1 M) to make a control solution of MGO (5 mM) and BSA (1 mg/mL). Two mL of control solution was incubated (6 days) with or without bean extracts (1 mL) in phosphate buffer [48–52]. Inhibition (%) of formation of AGE by extract for both models was calculated using the following equation:

### Measurement of tyrosinase inhibition activity

Microtiter plates (96-well) were used to perform assays and absorbance was measured (475 nm) by a plate reader. Each well contained sample (40 μL) and phosphate buffer (pH 6.8, 80 μL 0.1 M), tyrosinase (31 units/mL, 40 μL) and L-DOPA (2.5 mM; 40 μL), the samples were incubated (37°C) for half an hour and results are shown in Table 10. A control was prepared having all ingredients except tyrosinase [48–52]. The tyrosinase inhibition percentage was calculated as follows:

### Statistical analysis

All experiments were performed in triplicate and values marked by same letter in same column are not significantly different *(P <* 0.05). Data are expressed as the mean ± standard deviation. Data were analyzed by using the “MSTATC” statistical computer package [57].
